# The Impact of Different Intensities of Physical Activity on Serum Urate and Gout: A Mendelian Randomization Study

**DOI:** 10.3390/metabo14010066

**Published:** 2024-01-19

**Authors:** Tangxun Yang, Shilin Bi, Xing Zhang, Mingyue Yin, Siyuan Feng, Hansen Li

**Affiliations:** 1 School of Physical Education, Xihua University, Chengdu 610039, China; 2 National Institute of Education, Nanyang Technological University, Singapore 637616, Singapore; 3 Department of Physical Education and Sport, Faculty of Sport Sciences, University of Granada, 18071 Granada, Spain; 4 School of Athletic Performance, Shanghai University of Sport, Shanghai 200438, China; 5 Laboratory of Genetics, University of Wisconsin-Madison, Madison, WI 53706, USA; 6 Institute of Sports Science, College of Physical Education, Southwest University, Chongqing 400715, China

**Keywords:** physical activity, gout, metabolic disorder, chronic disease, uric

## Abstract

Physical activity is a potential protective factor against gout, but the role of exercise intensity in this context remains unclear. To overcome the limitations of observational studies in causal inference, this study employed a two-sample Mendelian randomization approach to explore the impact of different genetically proxied/predicted intensities of physical activity on serum urate concentration and the incidence of gout. Our data related to physical activity, serum urate, and gout were obtained from the UK Biobank, the Global Urate Genetics Consortium (GUGC), and the FinnGen dataset, respectively. Walking was included as representative of typical low-intensity physical activity in the analysis, and the other two types were moderate and vigorous physical activities. The estimation methods we used included the inverse-variance-weighted (IVW) method, MR-Egger regression, weighted-median method, simple-mode method, and weighted-mode method. Sensitivity analyses involved Rucker’s framework, Cochran’s Q test, funnel plots, MR-PRESSO outlier correction, and leave-one-out analysis. We found suggestive evidence from the inverse-variance-weighted method that moderate physical activity was a potential factor in reducing the incidence of gout (OR = 0.628, *p* = 0.034), and this association became more substantial in our subsequent sensitivity analysis (OR = 0.555, *p* = 0.006). However, we observed no distinctive effects of physical activity on serum urate concentration. In conclusion, our study supports some findings from observational studies and emphasizes the preventive role of moderate physical activity against gout. Given the limitations of the existing datasets, we call for future reexamination and expansion of our findings using new GWAS data.

## 1. Introduction

Gout is an acute sterile inflammation primarily triggered by the deposition of monosodium urate crystals in joints [1,2]. Historically, gout was thought to be a disease of only the wealthy or royal class, and it was called the “disease of the kings.” However, the incidence of gout has largely increased in multiple countries and regions as rapid economic development has provided the general population with richer diets [3]. From 2010 to 2020, the global incidence of gout rose from 0.08% to 2–4% [4,5]. In addition to severe pain symptoms, gout itself may contribute to an increased risk of conditions such as cardiovascular disease [6], chronic kidney disease [7], and rheumatoid arthritis [8]. Currently, gout remains a challenging condition to manage. Its progressively rising incidence, coupled with various associated comorbidities, poses a considerable economic burden on both individuals and society. A survey indicated that, compared with non-gout individuals, those with gout experience a substantial increase in annual medical expenses, amounting to several thousand or even tens of thousands of dollars [9]. Additionally, reports suggest that in the United States alone, the annual economic burden caused by gout exceeds billions of dollars [10]. Consequently, gout emerges as a noteworthy issue in the global public health domain.

The pathogenic mechanisms of gout are still quite complex [11], and the deposition of serum urate is widely recognized as the primary risk factor for gout [12]. Currently, pharmaceutical treatments are widely employed to address the issue of elevated serum urate levels causing gout [13]. While these medicines show good short-term efficacy, long-term use may induce gastrointestinal reactions, skin rashes, systemic complications, and even renal failure [14,15]. Furthermore, such medications cannot prevent, halt, or reverse the progression of this complex disease [16]. Therefore, there is an urgent need for non-pharmacological preventive and therapeutic approaches at present. In behavioral medicine, improving diet, physical activity, and alcohol consumption habits is considered beneficial for the prevention and treatment of gout [17]. Among these, physical activity may play a particularly crucial role. On one hand, there is a close relationship between physical activity and obesity (one of the risk factors for gout); on the other hand, skeletal muscles can produce anti-inflammatory cytokines during exercise, helping to break the vicious cycle of chronic inflammation and thereby reducing the impact of gout [18]. To explore the preventive effect of physical activity on gout, many scholars have conducted large-scale surveys, such as cross-sectional studies observing the association between physical activity and serum urate levels as well as gout symptoms [19,20]. Their research primarily supports the positive role of physical activity.

To address the limitations of observational studies, randomized controlled trials (RCTs), considered the “gold standard” for causal inference, are crucial [21]. However, high-quality RCTs often require substantial sample sizes and rigorous randomization processes to effectively balance confounding factors. Therefore, alternative research methods have considerable value, and Mendelian randomization (MR) has emerged as a novel technique in this context. MR uses genetic variants as instrumental variables to explore causal relationships between other external factors, thus allowing us to re-evaluate the findings from observational studies. MR is based on the principle that if genetic variation (or genes) influences a modifiable risk factor, and this risk factor, in turn, affects the risk of a certain disease, then genetic variation should be associated with the risk of that disease [22]. Eventually, we can estimate the causal impact of modifiable risk factors on disease risk based on certain assumptions [22]. Since an individual’s genotype is fixed at the formation of the zygote, theoretically the association between genes and traits should not be confounded by environmental factors encountered during an individual’s later life history [23]. Furthermore, the random allocation of genetic variation during meiosis and random mating within populations can further balance confounding factors [23]. These advantages make MR an analog of RCTs and thus play a crucial role in causal inference.

As mentioned above, since the previous findings on the association between physical activity and gout were obtained from observational studies, they can be threatened by confounding, and the causality cannot be confirmed due to the nature limitation of this research design [24], especially considering that gout symptoms can reduce overall activity levels (which implies reverse causality) [25]. Moreover, due to the often long and unpredictable intervals between gout attacks, exploring the impact of physical activity through large-sample and well-controlled trials is challenging and resource-intensive. For these reasons, using the MR approach to identify the causal effect of physical activity on gout and its risk factor serum urate is essential and may offer additional evidence to form a triangulation with existing studies of different designs [26]. So far, there has been only very limited exploration in this regard [27], and the intensity of physical activity has not been specifically studied. Since the intensity of physical activity is believed to alter its benefits on gout, our study, in response, is designed to explore the differences. In general, this study aims to use MR design to validate the impact of different intensities of physical activity on serum urate concentration and the incidence of gout.

## 2. Materials and Methods

### 2.1. Research Design

We employed a two-sample MR design for causal association evaluation. The two-sample MR is a typical technique within the MR framework. It extracts genetic variant—exposure and genetic variant—outcome association information from different study cohorts of the same underlying population [28,29]. To control for population stratification bias, we focused on individuals of European ancestry only in the current analysis [30]. This study is based on publicly available, summary-level GWAS (Genome-Wide Association Studies) data, eliminating the need for informed consent and ethical approval. The study was conducted according to the STROBE-MR statement [31].

### 2.2. Data Source

Single-nucleotide polymorphism (SNP) refers to a genetic variant in which a single base pair in the DNA varies across the population at an appreciable frequency [31]. In this study, SNPs were used as instrumental variables (IVs) to perform MR analysis.

Our “SNP—physical activity” data were obtained from the UK (United Kingdom) Biobank. The UK Biobank is a very large, population-based prospective study, established to allow detailed investigations of the genetic and nongenetic determinants of diseases [32]. In this survey, a series of questions were posed regarding participants’ physical activity habits. The question was, “On how many days per week do you respectively engage in walking, moderate physical activity, or vigorous physical activity that lasts 10 min?“ Responses were self-reported by participants and were coded as ordered categorical variables (0–7 representing 0 to 7 days per week). To our knowledge, this survey was modified from a well-studied three-item physical activity assessment tool [33,34]. It includes walking as representative of low-intensity physical activity, which may enhance the questionnaire’s applicability, as walking is a typical low-intensity leisure activity [35].

Our “SNP—serum urate” data were derived from a meta-analysis conducted by the Global Urate Genetics Consortium (GUGC). This meta-analysis combined data from 48 genome-wide association studies, establishing associations between genes and serum urate concentration (mg/dL). The study encompassed a cohort of 110,347 individuals of European ancestry [36].

Our “SNP—gout” data come from the FinnGen project in Finland. The FinnGen project, initiated in 2017, is a research endeavor aimed at collecting biological samples from 500,000 participants in Finland over six years. The project seeks to enhance health conditions through genetic research. Our “SNP—gout” data record the associations between genetic variants (SNPs) and the incidence of gout among participants. The outcome is encoded as a binary variable to represent the incidence of gout. All the data we utilized, along with their identification numbers and specific details, can be found in Table 1 of the IEU GWAS OPEN database [37].

### 2.3. Mendelian Randomization

**Figure 1:**
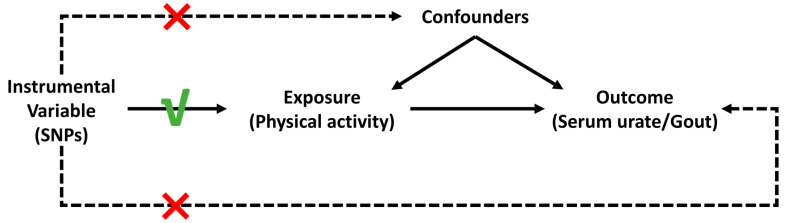
The assumptions of this Mendelian randomization study.

There are three fundamental assumptions when applying MR [38], which are that (1) the genotype is associated with the exposure; (2) the genotype is associated with the outcome through the studied exposure only (exclusion restriction assumption); and (3) the genotype is independent of other factors which affect the outcome (independence assumption). The assumptions applying to the current study are visualized in Figure 1.

To fulfill the first assumption, we used the conventional genome-wide significance threshold (*p* < 5 × 10^−8^) to choose genetic instruments that were strongly associated with exposures [39]. Moreover, all initially identified genetic variants were clumped using PLINK to ensure that our instruments came from an independent set of variants (settings: clump-r^2^ = 0.001 and clump-kb = 10,000) [40]. Then, data harmonization was performed to either correct or directly exclude the effects of ambiguous single-nucleotide polymorphisms (SNPs) with inconsistent alleles and palindromic SNPs with ambiguous strands [41]. The F-statistic for each instrument was estimated using F = beta^2^/SE^2^ [42,43,44]. IVs with an F-statistic smaller than 10 were considered weak instruments [45] and were excluded before analysis.

Unlike the first hypothesis, the other two MR hypotheses are unlikely to be fully validated [46,47]. On the one hand, the pleiotropy of genes is widespread. On the other hand, accurately specifying all confounding factors and excluding relevant SNPs is challenging. Therefore, we implemented several countermeasures to address this issue. First, although not always necessary, reverse causality between exposure and outcome may introduce bias to the estimation [23]. Considering this, we employed the Steiger filtering method to eliminate any SNP that was more predictive of the outcome than the exposure [48,49]. This method involves measuring the variance explained in exposure and outcome by SNPs, and testing whether the variance in the outcome is less than the exposure [50].

Secondly, horizontal pleiotropy is arguably the greatest threat to MR. It occurs when an SNP affects the confounders of the exposure—outcome association and eventually affects the outcome (referred to as correlated pleiotropy), or directly influences the outcome (referred to as uncorrelated pleiotropy) [51,52]. These two types of horizontal pleiotropy represent the violation of the second and third assumptions mentioned above [51]. For these reasons, evaluating and controlling for horizontal pleiotropy is highly important during MR analysis. Here, we mainly employed the MR-Egger intercept test and the “global test” of the MR Pleiotropy Residual Sum and Outlier test (MR-PRESSO) [53,54].

The MR-Egger intercept test is used to evaluate directional horizontal pleiotropy. Under the InSIDE (Instrument Strength Independent of Direct Effect) assumption, the intercept from the MR-Egger analysis can be interpreted as the average horizontal pleiotropic effect of the IVs included in the analysis, and an intercept not significantly different from zero can be interpreted as a balanced horizontal pleiotropy. Otherwise, there can be directional horizontal pleiotropy or a violated InSIDE assumption or both [53]. The MR-PRESSO global test evaluates the overall horizontal pleiotropy among all IVs in a single MR test by comparing the observed distance of all the variants to the regression line (residual sum of squares) with the expected distance under the null hypothesis of no horizontal pleiotropy [51]. In this study, a total of 10,000 simulations were computed to calculate the empirical *p*-values for the MR-PRESSO tests [51,55].

Three estimators were used for MR analyses, including inverse-variance weighting (IVW), MR-Egger regression, and weighted median. IVW was our core estimator and is the most efficient estimator [39] that can provide unbiased estimates under balanced horizontal pleiotropy [56]. We used the random effects model only for the IVW method as it tolerates balanced horizontal pleiotropy [57]. The MR-Egger is robust to directional horizontal pleiotropy under the InSIDE assumption, which allows for uncorrelated pleiotropy [28,52,58]. The weighted-median method can accommodate correlated horizontal pleiotropy [52] but requires that over 50% of IVs are valid [59]. We also considered two extra estimators, namely, simple and weighted mode estimators. The weighted mode-based estimators assume that the most common causal effect is consistent with the true causal effect. Hence, the remaining instruments could be invalid (that is, violate the assumptions of MR) without biasing the estimated causal effect [60,61]. A consistent direction of effect across all three methods strengthens the causal evidence, as each estimator makes different assumptions about pleiotropy [26,62].

### 2.4. Sensitivity Analysis

Since both the MR-Egger and MR-PRESSO rely on the InSIDE assumption [51,53] that is difficult to verify [63], we further supplemented two methods to check the pleiotropy issue. Firstly, we employed a funnel plot based on the core estimator used to visually assess overall directional pleiotropy. A symmetric funnel plot provides evidence for no horizontal pleiotropy or balanced horizontal pleiotropy [64,65]. Secondly, we conducted the Cochran Q test to assess heterogeneity [66]. While pleiotropy is not the sole cause of heterogeneity, it often leads to significant levels of heterogeneity [57]. Additionally, based on the Cochran Q test, we utilized the Rucker framework to examine whether our core estimator, IVW, provided a more optimal estimate. This framework calculates the change in Q value between IVW based on fixed and random models and MR-Egger estimates, thus suggesting the most reliable estimation [57].

After that, we used MR-PRESSO to address pleiotropy issues. MR-PRESSO is an IVW variant [39,41] that adjusts estimates based on detected pleiotropic instruments [51]. Finally, we performed a leave-one-out (LOO) analysis based on the IVW estimator to assess whether the results were altered by individual instrumental variables [57,67].

All statistical analyses were conducted using the TwoSampleMR (v.0.5.6), Mendelian Randomization (v 0.6.0), and MRPRESSO (v. 1.0) packages in R (v. 4.2.1). Due to multiple testing, we employed Bonferroni correction for statistical significance thresholds (0.050/3 exposures/2 outcomes = 0.008). A *p*-value less than 0.008 was deemed statistically significant, while *p*-values between 0.008 and 0.050 were considered suggestive evidence supporting causal associations [68,69].

## 3. Results

### 3.1. Horizontal Pleiotropy Assessment

We removed SNPs with stronger predictive power for the outcome variables using Steiger filtering. All the SNPs then exhibited ideal F-statistics (F > 10) and were therefore included in the analysis. The global test of MR-PRESSO revealed significant horizontal pleiotropy in all associations, except for the “moderate physical activity—gout” and “vigorous physical activity—serum urate” associations (Table 2), which justified the re-analysis after removing outliers (see Section 3.2 for sensitivity analysis). However, the MR-Egger intercept test only detected significant directional pleiotropy in the “moderate physical activity—serum urate” and “vigorous physical activity—gout” associations.

### 3.2. Mendelian Randomization

Regarding serum urate, MR-Egger regression showed that moderate physical activity was significantly associated with lower levels of serum urate (B = −1.520; *p* < 0.008) (Table 3). However, this result was inconsistent with the IVW estimate (*p* = 0.096). Apart from this association, all other associations did not reach statistical significance (*p* > 0.008), and there was no suggestive evidence found at a relaxed significance threshold (*p* > 0.05).

Regarding the impact of physical activity on gout, we did not find any associations reaching a significant level (*p* < 0.008) (Table 4). However, at the relaxed significance level, MR-Egger regression suggested that vigorous physical activity reduces the odds of gout (OR = 0.007; *p* = 0.030), but this result was not supported by our main estimator, IVW. On the other hand, the IVW method identified suggestive evidence supporting the association between moderate physical activity and gout incidence (OR = 0.628, *p* = 0.034).

### 3.3. Sensitivity Analysis

**Figure 2:**
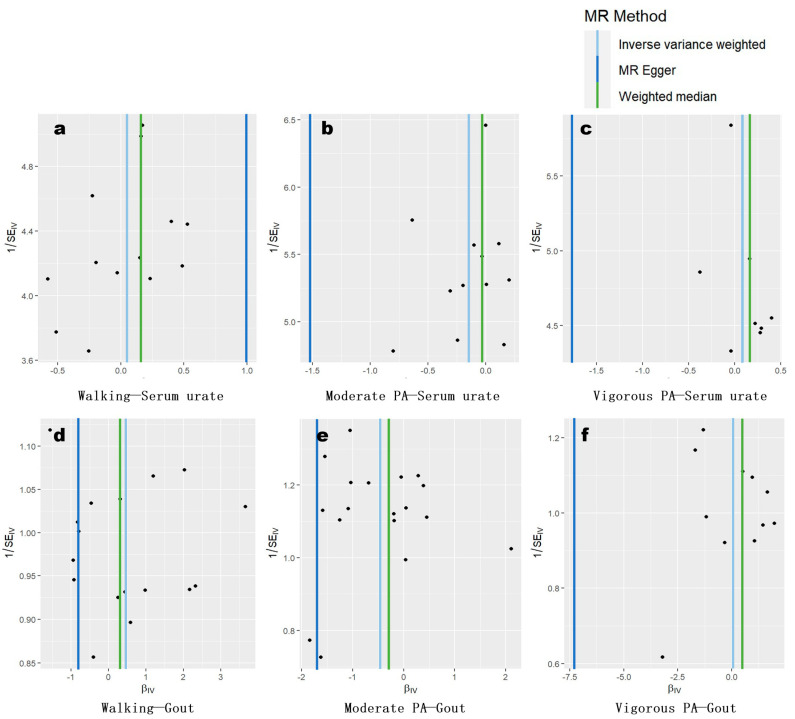
Funnel plots based on our core estimators. The *x* axis represents MR estimates, indicated by regression coefficients. The subfigures (**a**–**c**) indicate associations of walking, moderate PA, and vigorous PA with serum urate, while (**d**–**f**) indicate associations of walking, moderate PA, and vigorous PA with gout incidence.

Our funnel plots (Figure 2) indicated that MR-Egger regression exhibited bias in almost all analyses. In contrast, IVW performed the best among all estimators. For the observed potential association of “moderate physical activity—gout”, the scatter plot was generally symmetrically distributed around the reference line of IVW, suggesting a low likelihood of interference from directional pleiotropy in this result.

The Cochran Q test indicates that more than half of the IVW estimates are accompanied by significant heterogeneity (Table 5). In contrast, MR-Egger regression performed better in controlling heterogeneity, with significant heterogeneity found only in the analysis of “walking—serum urate” and “walking—gout” associations. Nevertheless, the potential causal association suggested by IVW in “moderate physical activity—gout” is not disturbed by heterogeneity (Q = 18.682, *p* = 0.347), further suggesting a low likelihood of interference from directional pleiotropy in this result. Based on the Cochran Q statistic, the Rucker framework was used to assess the appropriateness of the IVW results. The results indicated that, except for the associations of “moderate physical activity—serum urate” and “vigorous physical activity—gout”, which were more suitable to reference MR-Egger regression results, the other association results should be referred to IVW estimates. Additionally, the results suggest that, due to lower heterogeneity, the observed association of “moderate physical activity—gout” was more suitable for using the fixed-effects model of IVW estimation. Under this model, this association becomes more significant (OR = 0.628; *p* = 0.027).

The MR-PRESSO sensitivity analysis indicates that there were two and one outlier instrumental variables in the IVW estimates for the “moderate physical activity—serum urate” and “walking—gout” associations, respectively (Table 6). However, after removing these outliers, the previously observed associations were not substantially changed.

**Figure 3:**
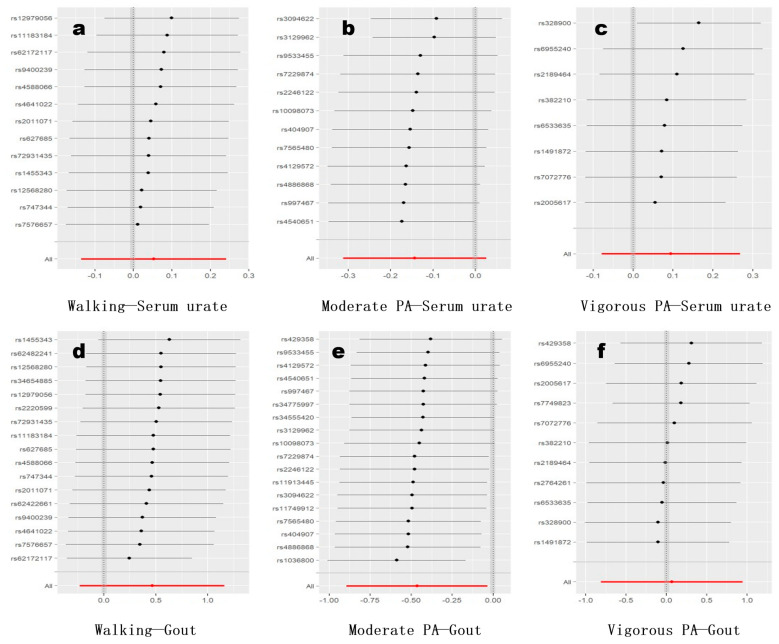
The leave-one-out analysis. The *x* axis represents the MR estimates, indicated by regression coefficients. The subfigures (**a**–**c**) indicate associations of walking, moderate PA, and vigorous PA with serum urate, while (**d**–**f**) indicate associations of walking, moderate PA, and vigorous PA with gout incidence.

Finally, we employed a leave-one-out method to assess the impact of each instrumental variable/SNP on the overall results (Figure 3). We found that the previously observed potential causal association between moderate physical activity and gout might be influenced by a single instrument variable (Figure 3e). After screening, we identified that the removal of rs1036800 resolved any contentious results in all re-analyses involving other instrument variables. Furthermore, after excluding this instrument, the “moderate physical activity-gout” association, estimated by the inverse-variance-weighted (IVW) method, reached statistical significance (OR = 0.555, *p* = 0.006). Importantly, none of the methods used for horizontal pleiotropy diagnostics (including MR-Egger intercept test, MR-PRESSO global test, and Cochran Q test) showed significant evidence of pleiotropy after removing this instrument, indicating the reliability of this result.

## 4. Discussion

This study aimed to employ MR approach to examine the impact of different intensities of physical activity on serum urate and gout. We obtained suggestive evidence from our core estimator (IVW) supporting the role of moderate-intensity physical activity in reducing the incidence of gout (OR = 0.628, *p* = 0.034). Moreover, none of the methods employed for detecting pleiotropy, including the MR-Egger intercept test, MR-PRESSO global test, funnel plots, and Cochran Q heterogeneity test, demonstrated evidence of horizontal pleiotropy that could distort this result. The Rucker framework used in our sensitivity analysis and a MR strategy by others [41] also support this result returned by IVW. Notably, after removing an instrumental variable that could lead to controversial results, this association reached the significance level set in our study (OR = 0.555, *p* = 0.006), further emphasizing the protective effect of moderate-intensity physical activity against gout. On the other hand, although MR-Egger regression suggested that moderate physical activity could reduce serum urate levels and vigorous physical activity could reduce the risk of gout, our funnel plot (based on MR-Egger) and pleiotropy diagnostics indicated that these results are possibly subject to horizontal pleiotropy. Therefore, we refrain from discussing these two associations further, focusing cautiously on the “moderate physical activity—gout” association only.

Our study results are generally consistent with some previous observational studies. For example, a cross-sectional survey in Sweden recruited 868 gout patients and compared them with randomly selected general participants, revealing lower levels of physical activity reported by male gout patients [19]. Another study showed that gout patients engaging in physical exercise had fewer annual episodes and reduced pain compared with those who did not exercise [70]. These studies collectively emphasize the protective role of physical activity against gout. However, since these studies only investigated a single time point, they cannot confirm the causal relationship between physical activity and gout. A study utilizing wearable devices for tracking physical activity identified a significant reduction in walking volume during gout attack periods, implying that patients with more frequent gout attacks may be less engaged in physical activity [71]. This uncertainty underscores the value of MR analysis since the causal direction is pre-determined in such a research design.

In recent years, scholars have increasingly employed this method to explore associations whose causality is challenging to determine through controlled experiments, such as the impact of physical activity on COVID-19 mortality [66], the influence of physical activity on cancer risk [72], and the effect of computer gaming on mental health [73]. Regarding the topic of gout, one relevant MR study indicated that the overall level of physical activity measured by accelerometers had no effect on the incidence of gout [27]. This finding contrasts with our “moderate physical activity—gout” association. We speculate that the different measurement methods of the independent variable (exposure) may be the primary reason for this discrepancy. The other study focused on accelerometer-measured physical activity, which is more objective and accurate, but it is difficult to distinguish the form and intensity of the physical activity. Thus, the measured physical activity might include portions unfavorable for reducing or even increasing the risk of gout, thereby weakening the strength of the association.

We did not find solid evidence to support the impact of physical activity on serum urate. Therefore, inflammation may be an important clue to explain our findings regarding gout. Research indicates that inflammatory factors such as interleukin (IL)-1β, IL-8, IL-17, NLRP3 inflammasome, and tumor necrosis factor-alpha (TNF-α) are involved in the inflammatory processes of gout, and immune cells, including neutrophils, monocytes/macrophages, and lymphocytes, play a crucial role in the onset of gout [74]. As mentioned in our introduction, myokines produced by physical activity help break the vicious cycle of chronic inflammation, thereby reducing the detrimental effects of gout [18]. Physical activity may exert anti-inflammatory effects by lowering IL-6, reducing C-reactive protein, and inhibiting TNF-α, thereby reducing the risk of gout [75,76,77]. However, the role of physical activity intensity in this regard is less understood. A controlled experiment on mice suggested that moderate-intensity exercise produced anti-inflammatory effects, while high-intensity exercise showed no significant difference in inflammation compared with the non-exercise control group [78]. This anti-inflammatory effect is believed to be achieved by physical activity through the downregulation of TLR2 on circulating neutrophils and inhibition of serum CXCL1 [78]. This result somewhat aligns with our study findings, indicating that the intensity of physical activity can modulate the protective effects of physical activity against gout. Nevertheless, since our study only analyzed associations and no other mediators were investigated, the specific mechanisms remain to be explored in future research.

This study has several advantages. Firstly, the MR approach allowed us to overcome the confounding effects between the exposure and outcome, regardless of whether the confounders were measured [79]. Secondly, we used distinct samples, avoiding the bias introduced by sample overlap in previous studies [29,80].

However, our study has some limitations. Firstly, the two-sample MR approach requires data from the same underlying population but different samples [29]. This is the reason why the STROBE-MR statement recommends a justification of the similarity of the genetic variant—exposure associations between the exposure and outcome samples [31]. However, since our exposure phenotypes (physical activity of different intensities) were not measured in the outcome database, we could not conduct such a comparison. Secondly, geographic clustering of genetic variation may introduce false genetic associations between our exposure and outcome. For example, mating behaviors (e.g., assortative mating) may be influenced by spatial factors. Therefore, individuals living in geographically close areas may have similar genes, leading to associations between genes and cultural, economic, social, political, and other environmental factors [81]. To address this issue, stratified analyses by region/area are necessary. However, the nature of the used summary data forbids such sensitivity analyses, which warrants further research.

## 5. Conclusions

This study aimed to use MR analysis to explore the impact of different intensities of physical activity on serum urate and gout. We found that moderate physical activity has the potential to reduce the incidence of gout, but we did not find conclusive evidence supporting the impact of physical activity on serum urate. Based on our findings, the development of exercise prescriptions involving moderate physical activity may contribute to preventing gout attacks. Future research should further investigate the mechanisms behind the ability of physical activity intensity to manage gout.

## Figures and Tables

**Table 1:** Data sources and IDs.

GWAS-ID	Phenotype	Sample Size	SNPs (*n*)	Ancestry
ukb-b-4886	Walking	454,783	9,851,867	European
ukb-b-4710	Moderate PA	440,266	9,851,867	European
ukb-b-151	Vigorous PA	440,512	9,851,867	European
ieu-a-1055	Serum urate	110,347	2,450,548	European
finn-b-M13_GOUT	Gout	150,797 (3576 cases and 147,221 controls)	16,380,152	European

Note: PA, physical activity.

**Table 2:** Horizontal pleiotropy assessment.

Exposure	Outcome	MR-PRESSO	MR-Egger	
		*p*	Intercept	*p*
Walking	Serum urate	0.010	−0.024	0.416
	Gout	0.008	0.033	0.770
Moderate PA	Serum urate	0.004	0.011	0.002
	Gout	0.370	0.042	0.373
Vigorous PA	Serum urate	0.207	0.051	0.084
	Gout	0.016	0.213	0.028

Note: PA, physical activity.

**Table 3:** Effects of physical activity on serum urate concentration.

Exposure	Estimator	B (95% CI)	*p*
Walking	MR-Egger	0.996 (−1.200, 3.793)	0.393
	Weighted median	0.156 (−0.037, 0.350)	0.113
	IVW	0.053 (−0.136, 0.240)	0.584
	Simple mode	0.186 (−0.181, 0.552)	0.341
	Weighted mode	0.177 (−0.162, 0.515)	0.236
Moderate PA	MR-Egger	−1.520 (−2.194, 0.847)	0.001
	Weighted median	−0.031 (−0.192, 0.129)	0.704
	IVW	−0.143 (−0.312, 0.025)	0.096
	Simple mode	−0.014 (−0.249, 0.221)	0.907
	Weighted mode	−0.011 (−0.223, 0.202)	0.923
Vigorous PA	MR-Egger	−1.756 (−3.515, 0.002)	0.098
	Weighted median	0.166 (−0.032, 0.364)	0.100
	IVW	0.094 (−0.079, 0.268)	0.286
	Simple mode	0.247 (−0.071, 0.566)	0.172
	Weighted mode	0.226 (−0.103, 0.555)	0.221

Note: B, standardized regression coefficient; CI, confidence interval; PA, physical activity.

**Table 4:** Effects of physical activity on gout incidence.

Exposure	Estimator	OR (95% CI)	*p*
Walking	MR-Egger	0.451 (0.0001, 1858.276)	0.854
	Weighted median	1.343 (0.629, 2.868)	0.446
	IVW	1.592 (0.792, 3.202)	0.192
	Simple mode	0.734 (0.177, 3.044)	0.675
	Weighted mode	0.681 (0.166, 2.797)	0.601
Moderate PA	MR-Egger	0.183 (0.013, 2.653)	0.231
	Weighted median	0.735 (0.409, 1.320)	0.303
	IVW	0.628 (0.409, 0.967)	0.034
	Simple mode	0.948 (0.341, 2.633)	0.920
	Weighted mode	0.936 (0.348, 2.516)	0.897
Vigorous PA	MR-Egger	0.0007 (2.59 × 10^−6^, −0.177)	0.030
	Weighted median	1.634 (0.621, 4.298)	0.320
	IVW	1.072 (0.446, 2.578)	0.877
	Simple mode	3.038 (0.561, 16.451)	0.227
	Weighted mode	2.793 (0.440, 17.752)	0.302

Note: OR, Odds Ratio; CI, confidence interval; PA, physical activity.

**Table 5:** Cochran Q test for heterogeneity.

Outcome	Exposure	MR-Egger		IVW	
		Q	*p*	Q	*p*
Serum urate	Walking	25.247	0.008	26.887	0.008
	Moderate PA	10.798	0.378	28.441	0.002
	Vigorous PA	5.716	0.456	10.002	0.188
Gout	Walking	33.300	0.004	33.497	0.006
	Moderate PA	17.749	0.339	18.682	0.347
	Vigorous PA	12.929	0.166	22.746	0.011

Note: PA, physical activity.

**Table 6:** MR-PRESSO outlier-corrected analyses.

Outcome	Exposure	Outlier (*n*)	Adj-ES	*p*
Serum urate	Walking	0	N.A.	N.A.
	Moderate PA	2	−0.036	0.506
	Vigorous PA	0	N.A.	N.A.
Gout	Walking	1	0.246	0.437
	Moderate PA	0	N.A.	N.A.
	Vigorous PA	0	N.A.	N.A.

Note: N.A., not applicable; Adj-ES, corrected effect size.

## Data Availability

Data are available from the sources stated in the main text.
